# Peer group-based online intervention program to empower families raising children with disabilities: protocol for a feasibility study using non-randomized waitlist-controlled trial

**DOI:** 10.1186/s40814-022-01190-1

**Published:** 2022-11-02

**Authors:** Akemi Matsuzawa, Rie Wakimizu, Iori Sato, Hiroshi Fujioka, Kaori Nishigaki, Seigo Suzuki, Naoko Iwata

**Affiliations:** 1 Department of Pediatric Nursing, Graduate School of Health Sciences and Faculty of Medicine, Hokkaido University, Sapporo, Hokkaido Japan; 2 Department of Child Health Care Nursing, Division of Health Innovation and Nursing, Institute of Medicine, University of Tsukuba, 1-1-1, Tennodai, Tsukuba, Ibaraki, 305-8575 Japan; 3 Department of Family Nursing, School of Health Sciences and Nursing, Graduate School of Medicine, The University of Tokyo, Tokyo, Japan; 4 Department of Nursing, Faculty of Health Sciences, Ibaraki Prefectural University of Health Sciences, Ami, Ibaraki Japan; 5 Department of Child Health Nursing, Graduate School of Nursing Sciences, St. Luke’s International University, Tokyo, Japan; 6 Department of Pediatric Nursing, Tokyo Medical University, Tokyo, Japan; 7 University of Tsukuba Hospital, Medical Liaison and Patient Support Center, Tsukuba, Ibaraki Japan

**Keywords:** Disabled children, Family, Family empowerment, Caregiver burden, Health care service, QOL, Peer group, Online program, Non-randomized waitlist-controlled trial

## Abstract

**Background:**

Families raising children with disabilities assume risks to their health and lives. Therefore, it is necessary to support these families to improve family empowerment, which is the ability of these families to control their own lives and to promote the collaborative raising of children with disabilities. This is the first online intervention program focusing on the empowerment of families raising children with disabilities who live at home in Japan.

**Method:**

The program consists of four online peer-based group sessions. Moreover, the families engage in several activities in stages wherein they discover their own issues, find measures to resolve them, and take action, while visualizing interfamily relationships, including social resources, and the status of their family life, with facilitators and other peer members. This study is a non-randomized, waitlist-controlled trial. It compares the results of the intervention group (early group) and the waitlist-controlled group (delayed group). The participants are allocated to the early or delayed group in the order of their applications. The main outcome is family empowerment. Other outcomes are the caregiver burden, self-reported capability to use social resources, self-compassion, and the quality of life (QOL) of primary caregivers. The timeline of the online outcome evaluation is as follows: the initial evaluation (Time 1 [T1]) is conducted before the start of the first early group program, and post-intervention evaluation (Time 2 [T2]) is conducted immediately (within 1 week) after the early group completes all four sessions (4 weeks) of the program. Follow-up evaluation (Time 3 [T3]) is conducted 4 weeks after the post-intervention evaluation. This timing is the same in the delayed group, but the delayed group will attend the program after a 4-week waiting period, compared to the early group.

**Discussion:**

The intention is to evaluate whether the provision of the program developed in this study and the evaluation test design are feasible and to verify the efficacy of this program.

**Trial registration:**

The UMIN Clinical Trials Registry (UMIN000044172), registration date: May 19, 2021.

**Supplementary Information:**

The online version contains supplementary material available at 10.1186/s40814-022-01190-1.

## Background

With highly advanced medical treatment enabling lifesaving techniques and shortened hospital stays, there are an increasing number of children with severe disabilities who are cared for at home, from childhood. In Japan, there has been a rapid increase in the number of children with severe disabilities who need constant medical care. According to the Ministry of Health, Labor and Welfare, the number of children with severe disabilities aged 0–19 years who need constant medical care was 9403 in 2005, and this number has doubled to 20,155 in 2020 [1].

Families raising children with disabilities face much higher risks to their own health and lives, compared to families raising healthy children. The mother, who is usually the primary caregiver, carries the burden of time and responsibility associated with providing the required care to the child and thus tends to have poor mental health [2]. Thus, there is an urgent need for direct support for families raising children with disabilities.

The concept of empowerment began to be used in the field of health sociology since the 1980s in the USA and from the 1990s in the UK [3, 4]. Family empowerment is the ability of families to control their own lives and to promote the collaborative raising of children with disabilities [5]. Collaborative partners who might be involved in raising children include other family members, medical and social service professionals, school teachers, and government officials. Koren et al. proposed a conceptual framework of empowerment for families raising children with disabilities in the community. This framework consists of “empowerment of family (internal) relationships,” “empowerment of relationships with service systems,” and “empowerment of interactions with the community” [6]. In Japan, the concept of empowerment was introduced in 1999 in the Guidelines for Support Services, including Care for People with Disabilities [7], and the concept of “family as a whole” for children with disabilities and their families was presented.

The mental health of mothers raising children with disabilities is related to their empowerment. Previous research has shown that depressive symptoms are associated with lower empowerment among mothers raising children with disabilities [8]. Also, mothers raising children with developmental disabilities participated in health-promoting activities infrequently; moreover, depressive symptoms, maternal empowerment, and two indicators of child-related QoL explained significant variance in healthy behaviors [9]. Parents often face a variety of difficulties in raising children with disabilities. Therefore, these parents need to be able to not only receive social resources including healthcare services but also use and coordinate social resources that are necessary for their children and families. In addition, these parents need the skills to cope with child- and family-related problems.

In a previous study, we conducted in-depth interviews with 34 families with children with severe disabilities who lived at home (mother, father, and siblings aged 12 years and older) [10] and conducted a questionnaire survey of 158 nurses and government officials using the Delphi method [11] to comprehensively list the factors that comprise the family empowerment model. Thereafter, we issued a self-administered questionnaire based on that model to 1659 families nationwide with children with severe disabilities who were living at home, to develop and verify the family empowerment model [12]. This research undertaken with family caregivers has shown an association between the empowerment and knowledge of social resources and how to use these resources in order to reduce burden. However, the above were observational studies. In this study, we verify, through implementational research, whether an intervention for each of these factors (specifically, by working on increasing the “self-reported capability to use social resources” and reducing the “caregiver burden”) can actually increase family empowerment. Furthermore, we aim to determine which of the three levels of Koren et al.’s conceptual framework (“family [internal] FA”, “service system SS,” and “community SP”) are actually empowered by this intervention.

### Purpose

This paper describes the development and implementation of a peer group-based online intervention program to empower families raising children with disabilities. The primary objective of this study is to explore the feasibility of delivering a non-waitlist randomized controlled trial to a group-based intervention program of the “Family Empowerment Program of families raising children with disabilities” in the community. The secondary objective is to determine whether the peer-led, online group-based Family Empowerment Program for families raising children with disabilities is more effective at empowering families and reducing the caregiver burden and improving the self-reported capability to use social resources, self-compassion, and health-related quality of life (HR-QOL) of families raising children with disabilities. We have developed and now plan to evaluate, the first online program designed to empower families caring for children with disabilities who live at home, in Japan.

## Method

### Study design and setting

**Fig. 1:**
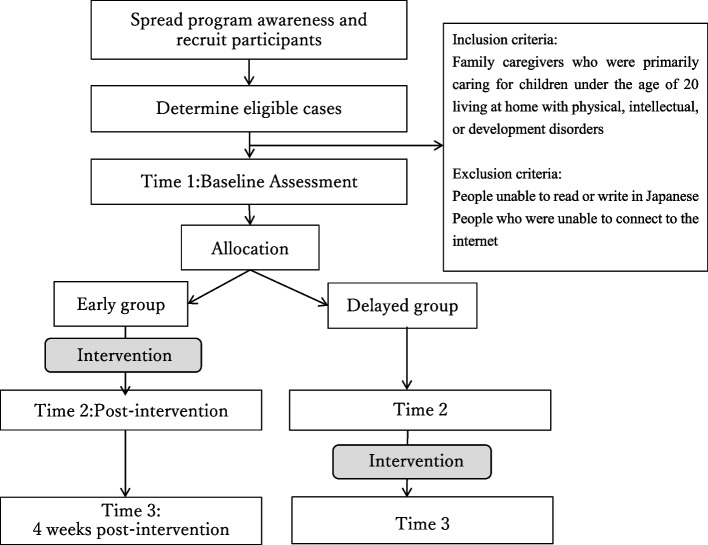
Trial flow chart

**Fig. 2:**
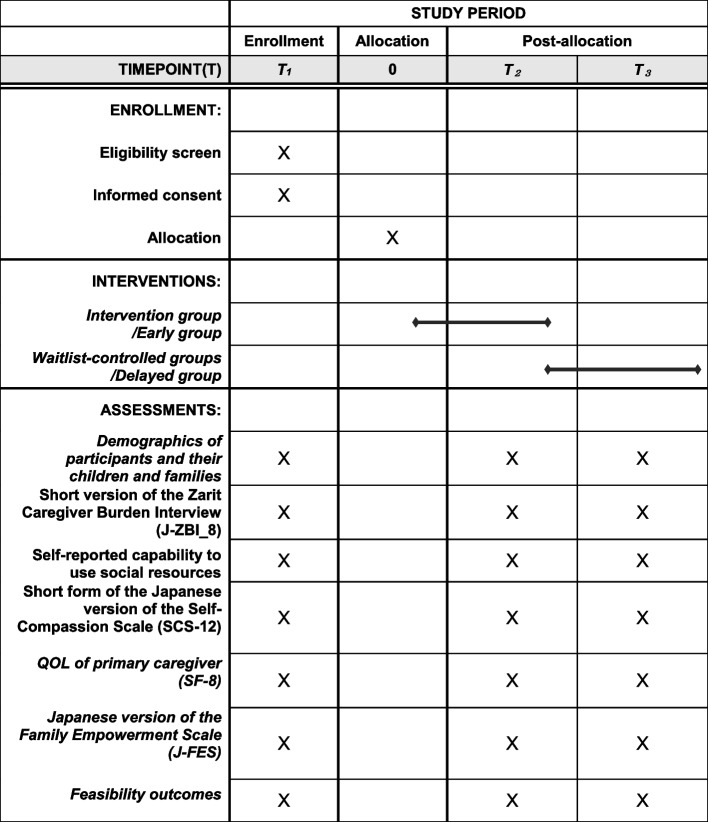
The SPIRIT schedule of enrollment, interventions, and assessments

This study is a non-randomized, waitlist-controlled trial. We compare the group starting the family empowerment program a week after the initial evaluation (intervention/early group) to the group starting delayed intervention (waitlist-controlled/delayed group). This study is registered as a clinical trial in the UMIN Clinical Trials Registry (UMIN000044172). The study protocol has been prepared in accordance with the Standard Protocol Items: Recommendations for Intervention trials (SPIRIT) 2013 statement [13]. The trial and flow of participants through the trial are illustrated in the flow chart (Fig. 1). The SPIRIT schedule of enrollment, interventions, and assessments are presented in Fig. 2. The SPIRIT Checklist can be found in Additional file 1. This protocol is version 1, dated June 2021.

### Recruitment methods

A written request for cooperation in the study is sent by post to people in charge of medical institutions, government agencies, and special-needs schools nationwide. The organizations that are requested to cooperate are specialized medical institutions and rehabilitation centers for children with physical disabilities, university hospitals and specialist pediatric hospitals, medical welfare departments of local governments, and special-needs schools for children with physical impairment or chronic diseases.

People in charge of organizations who consent to cooperate in the study are requested to display posters to recruit program participants within their facility and distribute flyers explaining the research to people who match the inclusion criteria and do not fall under the exclusion criteria. The flyers describe the general outline of the family empowerment program, the schedule, target participants, and implementation method. People interested in the study are requested to access a dedicated website from the information in the flyer and apply to participate in the study after consenting to the content of the study’s flyer. To reach the target sample size, requests for study cooperation may also be sent to associations for families with disabled children and day care centers for children with disabilities, depending on the status of participant registration.

### Inclusion criteria and exclusion criteria

Target participants are family caregivers who are primarily caring for children under the age of 20 with physical, intellectual, or developmental disorders, at home. Exclusion criteria include people who are unable to read or write in Japanese, and those who are unable to connect to the Internet.

### Sample size

The sample size is estimated by selecting *F* tests (ANCOVA) using G*Power software version 3.1.9.7 [14]. The required sample size is calculated based on data obtained from the pretest implemented with the same subjects and equivalent program content as used in this study. The required sample size is 52, assuming an effect size of 0.33, a test power of 80%, and an *α* value of 0.05. The dropout rate is assumed to be 10% based on a previous study with an online program for parents of children with disabilities [15]. From a feasibility study perspective, a formal sample size calculation is not essential. Our sample size calculation is based on the pragmatics of recruitment and the necessity for examining feasibility among a broad group of families caring for children with disabilities.

### Intervention allocation

Participants are allocated to the early or delayed group in the order of their applications. A maximum of 20 people can participate in each program, and the family empowerment program comprising a total of four group sessions is implemented several times per month. Participants in both groups will not be told the timings of the intervention or the evaluations in the other group in an attempt to keep all participants blinded as to which group they are allocated to. Facilitators of the family empowerment program cannot be blinded to the participants’ allocations because they need to contact the participants and respond to inquiries. The analysts are blinded to the allocation of participants.

### Reducing the risk of participant drop-out

To reduce the risk of participant drop-out, we ensure that participants can contact the principal investigator directly by email or telephone with any questions about participation before enrolling in the program. In addition, after deciding to participate, participants receive individual confirmation emails, with information regarding the arrival of the program textbook and guidance on participation methods, before the program’s start date. Each participant is also sent reminders to respond to questionnaires required for program evaluation, depending on the available response timeframe and response status of each participant.

### Ethical considerations

The protocol for this study was reviewed and approved by the institutional review board established by the principal investigator’s affiliated institution. Voluntary written informed consent is obtained from the participants before the initial evaluation. The explanatory document for this study includes matters that need to be explained to the participants according to the “Ethical Guidelines for Life Science and Medical Research Involving Human Subjects” formulated by the Ministry of Health, Labor and Welfare. That is, it includes information on the purpose and significance of the study, the study methods, the burden on the study subjects, expected risks and benefits to the subjects, management of personal information, methods to store and dispose of information, and financial burden on the study subjects. The study subjects can freely choose to participate or abstain from the study without being disadvantaged in any way. Their declaration of intent to participate in the study may be withdrawn at any time without providing a reason. Moreover, participants’ personal information and data remain confidential. The data collected in this study, including the questionnaire responses, are stored for a certain period, even after the end of the study.

### Intervention methods

The intervention program for this study is developed using an intervention mapping approach [16]. We have developed a peer group-based support program based on the social cognitive theory [17], a taxonomy of behavior change techniques (BCTs), and a practical behavior change technique [18], as an intervention method to encourage changes in family behavior, with the aim of improving family empowerment in families raising children with disabilities who live at home. The described intervention has been further extracted using the “Better reporting of interventions: template for intervention description and replication (TIDieR) checklist and guide [19].” The specific development process for the intervention used in this study is described in a separate paper [20].

The program holds a total of four weekly group sessions every Saturday afternoon continuously for 1 month using an online meeting system (ZOOM®). Each session is scheduled for two hours. In each group session, participants are separated into two to four sub-groups (around three to six people per group) of smaller online rooms (breakout rooms), in line with the type of disability and the age of the child. During this time, participants share their opinions, questions, and answers. In every session, the discussion time of the online smaller rooms is for at least 60 min. At the start and the end of the session, all the participants gather together. At the end of the session, facilitators share their impressions of the session with the whole group. The participants receive a textbook by post approximately 1 week before the first program. Four to six researchers take turns as the program facilitators each time. The main facilitators are fixed for all four sessions, while three to four facilitators are responsible for encouraging sharing among participants in breakout rooms and following up with participants about the online meeting system.

The program details are as follows. In the first session, each participant creates and shares an eco-map [21] with the aim of “understanding the current circumstances surrounding the child and the family,” while reflecting upon their own lives and the lives of their disabled children and their families at the present time. Finding elements of family empowerment in each participant’s life leads to a basic understanding of family empowerment. Furthermore, participants’ sharing of their eco-maps in the group promoted mutual understanding among them and which may have the secondary effect of promoting group dynamics. In the second session, all participants share their homework, which is to create a table to record a week in their lives between the first and second sessions, with the aim of “reflecting on the child’s and the family’s actual life to clarify the participant’s desired life.” Time is set aside for participants to think about problems in their current lives and to imagine the kind of life they want for themselves and their families. In the third session, participants engage in a clear goal-setting exercise to achieve the kind of life they want for themselves and for their families and set specific behavior goals to achieve that end; they share this information with the group. Participants think of goals, such as “I would like to be able to work full time in the 3 years later”, or “I would like to take the whole family on an overnight trip in the next year.” Time is set aside to think in relation to each domain of family empowerment, which links “What can be done within the family? (FA),” “What can be done with service providers (SS),” and “What can be done with government officials in the local community? (SP).” In the fourth session, participants share their “efforts to work toward the life they want,” which is the homework between the third and fourth sessions; they also reflect on the group work as a whole.

Throughout the program described above, participants are reminded to set clear goals based on the characteristics of their families, on raising a child with disabilities, to promote self-monitoring, to provide information, to clarify barriers to benefits and risks related to health and life, to promote peer support, to promote self-compassion, and to promote awareness of family empowerment.

Interactions between parents and children at different developmental stages aim to provide the opportunity for parents with younger children to acquire information on their outlook for the future and for parents with older children to self-monitor by looking back on their progress to date. This program is based on peer groups; therefore, the facilitators devise ways to ensure that the participants’ initiative and interaction among members are demonstrated in the sessions. Specifically, facilitators try to create a relaxed situation by allowing participants to speak more and by allowing other members to freely express their opinions in response to members’ comments. This program is repeated over a period of about 6 months.

### Personal education tools

We prepared three types of tools: a workbook, a booklet on family empowerment as a sub-textbook, and an online meeting system manual. The textbook, booklet, and online meeting system manual are distributed to the program participants. The online meeting manual, which describes the use of Zoom® is distributed and made available online. The textbook was printed in color, and innovations were added, such as large font and large sheets of paper for homework, to ensure that the participants could comfortably participate in the program. A certificate of completion was inserted at the end of the textbook, and after participating in the last session, a PDF copy of the certificate of completion was emailed to the participants, with the aim of improving self-compassion. The booklet is used as a tool to promote awareness of family empowerment. An explanation of the three levels of family empowerment and explanations of each item were provided with illustrations, ensuring that the participants can easily refer to the content during the program and can link the content to family empowerment and their situations.

### Facilitators

The lead facilitators who oversee the entire program are university faculty members who have specialized in pediatric or family nursing with a career of about 10 years. Furthermore, when the participants are separated into smaller groups, the facilitators in charge of the smaller groups are people with deep ties to children with disabilities and their family, such as specialists routinely involved with these children, family members, or parties raising these children. A facilitation book is created to standardize the program. This book follows the textbook, describing in detail, the time needed for work within the program and important points to note, to ensure that the quality of progress is sustained among the facilitators.

### Data collection method

In order to evaluate the effectiveness of this program, data is collected from participants through an online survey. Participants are invited to complete online questionnaires that assess their family empowerment, caregiver burden, self-reported capability to use social resources, self-compassion, and quality of life. A survey request email is sent to the participants regarding the online survey at the times described below, and one or more reminders are sent to those who do not respond. Participants are requested to cooperate in the survey regardless of their program attendance. For example, even if a participant does not attend any sessions, they are asked to complete the survey.

The online survey is conducted at the following three time points. After the participants apply for the program and are allocated to either the early or delayed group, they are notified of their attendance month (from July to December). At the time, participants are not informed if they are in the early or delayed group; they are simply notified of their attendance month to prevent response bias based on the participant’s impression of their allocated group. Thereafter, the initial evaluation (Time 1 [T1]) is conducted before the early group starts the first session. For the early group, this corresponded roughly to a week before attending the program. The post-intervention evaluation (Time 2 [T2]) is conducted immediately (within 1 week) after the early group completed all four sessions in the program (4 weeks). The follow-up evaluation (Time 3 [T3]) is conducted 4 weeks after the post-intervention evaluation. The timings are the same for the delayed group; however, it begins the program precisely 4 weeks after the early group does. That is, the delayed group completes the evaluation for T1 roughly 5 weeks before attending the program, T2 immediately before attending the program, and T3 immediately after completing the course.

The response information is collected in the online survey system. Participants are allocated identity documents (IDs) that are linked to the response information, and every response of each participant from T1 to T3 can be linked. If there are multiple responses from the same person at the same time (duplications), it is assumed that it is because the respondent wished to correct their answer; hence, the later response is adopted. The data collected in the online survey system are backed up fortnightly. Once all the participants complete the follow-up evaluations, the data are downloaded from the system and stored as primary raw data.

### Outcomes and measurement methods

### Feasibility outcomes

The primary outcome of this study is to determine the feasibility of a future study. Feasibility will be assessed through recruitment and retention rates assessed at pre-intervention through to T3. Acceptability will be assessed through participant feedback on satisfaction and adverse events with the conduct of the intervention collected at T3.

### Main outcome

The main outcome is family empowerment. Family empowerment is measured each time at T1–T3 using the Japanese version of the Family Empowerment Scale (FES) [22]. FES evaluates whether a family is able to control its family life while collaborating with others, addressing the three levels of “Family (internal) relationships (FA),” “Relationships with service systems (SS),” and “Involvement with community (SP).” Responses to 34 items on a 5-point scale are summed, and the results are obtained as the total score and three subscale scores. The higher the score, the higher the level of family empowerment. In case of missing responses, if more than half of items are answered, the missing data are supplemented by averaging the answered items.

Secondary outcomes are caregiver burden, self-reported capability to use social resources, self-compassion, and QOL of primary caregivers. Caregiver burden is measured using the short form of the Japanese version of the Zarit Caregiver Burden Interview (J-ZBI-8) [23]. The ZBI-8 evaluates caregiver burden, including physical and mental burden and constraints on social activities. Eight items are answered on a 5-point scale, and the caregiver burden score is obtained as the total score by summing the responses. The higher the score, the greater the caregiver burden. These outcomes are evaluated each time at T1–T3. In case of missing responses, if more than half of items are answered, the missing data are supplemented by averaging the answered items.

It has been shown that parents raising children with disabilities have sleep disturbances, which creates a burden for parents [24]. Therefore, we added two items on sleep-related issues. One question inquires whether the parent gets up during the night to care for the child: “How often do you need to get up during the night to care for your child with disabilities?” The respondents chose “every night,” “several nights a week,” “several nights a month,” “never,” and “other (state specific information).” The other question asks about the average hours of sleep per day. These items are asked each time at T1–T3.

Two original items are created regarding the use of social resources based on discussions among the researchers in our previous study [12]. The first question is asked each time at T1–T3: “Do you feel you can use social resources, properly?” and respondents can select from “I often use social resources,” “I sometimes use social resources,” “I do not use social resources very often,” and “I never use social resources.” The second question, asked at T2 and T3, is “Do you feel that you are now more able to properly use social resources compared to when you answered the previous questionnaire?” and the respondents selected “can no longer use social resources,” “cannot use social resources very much,” “unchanged,” “can use social resources slightly more,” or “can use social resources very often.” The survey also inquires about the participants’ actual use of social resources (actual use volume and frequency) as basic attributes.

Self-compassion is measured each time at T1–T3 using the short form of the Japanese version of the Self-Compassion Scale (SCS) [25]. SCS is comprised of 12 items on a 5-point scale, and there are two items applicable to each of the following aspects of “self-kindness,” “self-judgement,” “common humanity,” “isolation,” “mindfulness,” and “over-identification,” and six-scaled scores are obtained by totaling the answers. The higher the score, the higher the level of self-compassion. In case of missing responses, if more than half of the items are answered, the missing data are supplemented by averaging the answered items.

The QOL of the primary caregiver is measured each time at T1–T3 using the SF-8 [26]. SF8 is an 8-item, 5–6-point scale, with scores calculated based on a distribution of 50 points as the standard value using a unique scoring algorithm. Missing data are also handled using this scoring algorithm. The higher the score, the higher the health-related QOL.

In addition to these outcomes, the following questions are asked about basic attributes. At T1, the participants are asked about their relationship (from the child’s perspective), age (in 10-year increments), marital status, cohabitation status with partner, highest level of education, occupation, household income, and family members living in the same household. At T1, the following questions are asked about the child’s attributes: gender, age, name of the diagnosis, age at diagnosis, school attendance history, condition of disability, and required care. At T2 and T3, the respondents are asked if there are any changes in these circumstances and are asked to provide details if there are any. To ensure that the responses are not compulsory, the respondents are given the option of, “No response” and the online survey system is arranged so that respondents could progress to the next page without answering some or all questions.

The respondents are asked the following questions in the questionnaire to evaluate the process after attending the program and four weeks later: How did you feel after attending the program? Did you tell your family about the content of the program and the information you learned? Would you recommend the program to your friends? How can the program be expanded? They are asked their opinion about the program (age of their child when they attended the program, number of sessions, days the sessions are held, the length of the program, and the necessity of childcare).

### Data analysis

For the assessment of feasibility, which is the primary outcome of this study, descriptive statistics will be used to assess the feasibility outcomes, such as the recruitment and retention rate of participants across the intervention. Intervention acceptability will be assessed through analysis of the level of satisfaction, opinions, and free responses related to the program described and listed.

This program is designed such that attending a total of four sessions enables participants to sequentially review their family situation including the relationships within their families and their social resources, their own, and their family members’ lives and to visualize their family relationships, burden, and the use of services. Specifically, after attending the program, participants are expected to have an improved sense of family empowerment immediately, including establishing and practicing specific and feasible plans. Therefore, to verify the effectiveness of the program, the primary outcome measure is the total FES score at T2. The secondary outcomes are to verify improvement in the levels of family empowerment (FES subscale score at T2), whether aspects directly addressed in the program had improved (caregiver burden, self-reported capability to use social resources, self-compassion at T2), and whether they had a comprehensive effect (health-related QOL at T2). Furthermore, whether any interventional effect observed at T2 is sustained (each outcome score at T3 for the early group), and whether any interventional effect observed in the early group is reproduced (each outcome score at T3 for the delayed group) is verified. The statistical analysis plan is described as follows:

The responses, wherein the outcome scores can be calculated as valid responses, are used. ITT analysis is performed to manage data from participants who deviate from the study protocol by not attending the program at the designated times as the original allocation group. First, descriptive statistics are calculated for the background attributes and outcome scores of the participants (both the participant and the child) at T1. The interval scale is used to calculate the mean and standard deviation; ordinal and nominal scales are used to calculate the frequency and proportion, and intergroup comparison is performed using Welch’s *t* test, the Mann-Whitney *U* test, and Fisher’s exact probability test.

There is a possibility of participants dropping out between T1 and T2 and between T2 and T3. Therefore, we compare the background attributes and outcome scores at T1 between the primary outcomes “total FES score of valid respondents at T2” and “non-respondents at T2 (but are valid respondents at T1)” to examine and discuss the effect caused by participants dropping out. In addition, an intergroup comparison of background attributes and outcome scores at T1 is conducted for a population of only the “total FES score of valid respondents at T2.” If group differences that may create significant bias or biased dropouts are observed in the analysis to date, we aim to consider adding those variables as covariates in subsequent analyses.

The primary analysis is an intergroup comparison using analysis of covariance with the total FES score at T1 as the covariate, and the total FES score at T2 as the dependent variable. In case of a significant interaction between the independent variable (group: early group or delayed group) and the covariate (total FES score at T1), an intergroup comparison is conducted using ANOVA without considering the scores at T1. The level of statistical significance is set as 5%. Interim analysis is not performed. Subgroup analysis is conducted for children with severe disabilities, children with disabilities who need constant medical care, and single-parent households. We also examine whether the increase in the total FES score from T1 to T2 is clinically significant. However, the clinical minimum significant difference for FES is not clear; therefore, this is discussed through a comparison with a previous study [12].

An intergroup comparison is similarly conducted for the secondary outcomes using the analysis of covariance for each outcome at T2. The results at T1 and T3 for the early group are examined using a paired *t* test to determine whether the intervention effect observed at T2 is sustained. The results at T2 and T3 for the delayed group are examined using a paired *t* test to determine whether the intervention effect observed in the early group is reproduced in the delayed group.

The following two analyses are conducted as a sensitivity analysis of the primary analysis. The first is a re-analysis of the dropouts from T1 to T2 with the amount of change in the total FES score set as 0, to consider the effect of dropouts. The second is a per-protocol analysis. That is, re-analysis is conducted to compare the results of participants who attended all four sessions of the program (group) to all others (group).

### Data management

All self-reported data is entered directly into a web-based data capture system via an online survey. Only the researchers on the team have access to the data set, and the data is password protected. We believe that this study is low-risk, and therefore, a data monitoring committee is not necessary.

## Discussion

We previously developed a peer-group-based online intervention program with the aim of empowering families to raise children with disabilities. There are few evidence-based programs in the literature that employ intervention programs for families raising children with disabilities [27, 28]. This is the first initiative for an evidence-based program for families raising children with disabilities at home, which is developed, implemented, and scientifically evaluated in Japan. This study provides a detailed protocol for the intervention program.

The family empowerment program we developed for families raising children with disabilities uses a participant-led approach, enabling participants to discover their own problems, and find and act on measures to resolve those problems through activities, while visualizing interfamily relationships, including social resources, and the current family situation. These peer group-based activities are performed in stages, and the expected outcomes for the participating families include improved empowerment, reduced caregiver burden, and improved utilization of social resources. An expected secondary effect of participation in a peer group-based program is the formation of social-support networks for these families.

Parents of children with disabilities are always busy raising and caring for these children and their siblings and are unable to get enough sleep or free time for themselves. Therefore, it is difficult for them to participate in intervention programs. However, we have improvised the content of the program such that it could be conducted online during the coronavirus disease 2019 (COVID-19) crisis. Consequently, this approach allows families to connect with other families located far away who are also raising children with disabilities, without leaving home. The participants are able to share their experiences of discovering their own problems and find measures to resolve these problems, thus increasing the feasibility of the program.

Converting the results of this study to an evidence-based community program and widely disseminating the program is a challenge. In the future, it will be necessary to verify the effectiveness of this program and to determine if there are any adverse effects of running pilot tests. It is also essential to improve the program based on the results of the verification.

## Supplementary Information

**Additional file 1.** SPRIT 2013 Checklist: Recommended items to address in a clinical trial protocol and related documents.

**Additional file 2.** The TIDieR (Template for Intervention Description and Replication) Checklist: Information to include when describing an intervention and the location of the information.

## Data Availability

Not applicable.

## Abbreviations

BCT: Behavior change technique
COVID-19: Coronavirus disease 2019
FA: Family (internal)
FES: Family Empowerment Scale
ID: Identity document
QOL: Quality of life
SCS: Self-Compassion Scale
SP: Community
SS: Service system
T1: Time 1
T2: Time 2
T3: Time 3
ZBI-8: Zarit caregiver burden interview
