# Supplementary material for: Peer group-based online intervention program to empower families raising children with disabilities: protocol for a feasibility study using non-randomized waitlist-controlled trial
Source: Pilot Feasibility Stud. 2022 Nov 2;8:233. doi: 10.1186/s40814-022-01190-1 (PMC9628164; doi:10.1186/s40814-022-01190-1)
Supplement: Supplementary file 2 — Additional file 2. The TIDieR (Template for Intervention Description and Replication) Checklist: Information to include when describing an intervention and the location of the information. [file 40814_2022_1190_MOESM2_ESM.docx]

**Additional File2: TIDieR Checklist of this study’s intervention**

| **Item num-ber** | **Items** | **Location** | |
| --- | --- | --- | --- |
|  |  | **Primary paper**  **(page)** | **Details** |
| **1.** | **BRIEF NAME**  Provide the name or a phrase that describes the intervention. | p.6 | The name of the program is the “Family Empowerment Program of families raising children with disabilities”. |
| **2.** | **WHY**  Describe any rationale, theory, or goal of the elements essential to the intervention. | p.8-13 | This study will be conducted as a peer group-based online program based on the concept of family empowerment and using Health Behavior Theory. The goal of the program is to empower families to take control of their lives and raise their children with disabilities. |
| **3.** | **WHAT**  Materials: Describe any physical or informational materials used in the intervention, including those provided to participants or used in intervention delivery or in training of intervention providers. Provide information on where the materials can be accessed (e.g. online appendix, URL). | p.13 | We prepared three types of tools: a workbook, a booklet on family empowerment as a sub-textbook, an online system manual, and facilitator’s manual. This program consists of four sessions. The text is divided into four chapters, and the themes of each chapter are as follows: First session, “understanding the current circumstances surrounding children and families”; Second session, “reflecting on the life of children and families, and clarifying the participants’ desired life”; Third session, “setting goals for the life that the children and families want”; and Fourth session, “reflecting on the group work”. All are written in Japanese. |
| **4.** | Procedures: Describe each of the procedures, activities, and/or processes used in the intervention, including any enabling or support activities. | p.10-12 | This program consists of a total of four sessions, with specific objectives for each session, and is based on small group discussions. |
| **5.** | **WHO PROVIDED**  For each category of intervention provider (e.g. psychologist, nursing assistant), describe their expertise, background and any specific training given. | p.13-14 | The facilitators who oversee the entire program are university faculty members who have specialized in paediatric or family nursing with a career of about 10 years. |
| **6.** | **HOW**  Describe the modes of delivery (e.g. face-to-face or by some other mechanism, such as internet or telephone) of the intervention and whether it was provided individually or in a group. | p.10-12 | The program held a total of four group sessions every Saturday using an online meeting system |
| **7.** | **WHERE**  Describe the type(s) of location(s) where the intervention occurred, including any necessary infrastructure or relevant features. | p.10 | This program uses an online meeting system. Therefore, participants can choose their home or any other location of their choice to participate in the program. |
| **8.** | **WHEN and HOW MUCH**  Describe the number of times the intervention was delivered and over what period of time including the number of sessions, their schedule, and their duration, intensity or dose. | p.10-13 | This program has a total of four sessions and is held every Saturday afternoon continuously for one month. Each session lasts for two hours. |
| **9.** | **TAILORING**  If the intervention was planned to be personalised, titrated or adapted, then describe what, why, when, and how. | N/A |  |
| **10.** | **MODIFICATIONS**  If the intervention was modified during the course of the study, describe the changes (what, why, when, and how). | N/A |  |
| **11.** | **HOW WELL**  Planned: If intervention adherence or fidelity was assessed, describe how and by whom, and if any strategies were used to maintain or improve fidelity, describe them. | N/A |  |
| **12.** | Actual: If intervention adherence or fidelity was assessed, describe the extent to which the intervention was delivered as planned. | N/A |  |
